# P-659. Active Surveillance of Mucosal Pneumococcal Infections to Predict the Potential Added Benefits of Extended-Spectrum PCVs in Children < 24 Months in Southern Israel

**DOI:** 10.1093/ofid/ofae631.856

**Published:** 2025-01-29

**Authors:** Ron Dagan, Rachel Benisty, Bart Adriaan van der Beek

**Affiliations:** Ben-Gurion University of the Negev, Beer Sheva, HaDarom, Israel; Soroka University Med Ctr and Ben-Gurion University, Beer Sheva, HaDarom, Israel; Ben-Gurion University of the Negev, Beer Sheva, HaDarom, Israel

## Abstract

**Background:**

Although mucosal infections, such as otitis media (OM) and acute conjunctivitis (AC) are the most common manifestations of pneumococcal disease in young children, surveillances have been based mainly on invasive pneumococcal disease (IPD) due to paucity of serotype-specific data on mucosal infection rates. We present rates and serotype dynamics of OM and AC as a means for assessing potential added benefits of the new PCVs, (PCV15/PCV20) against OM and AC in children < 24m.

**Figure:**
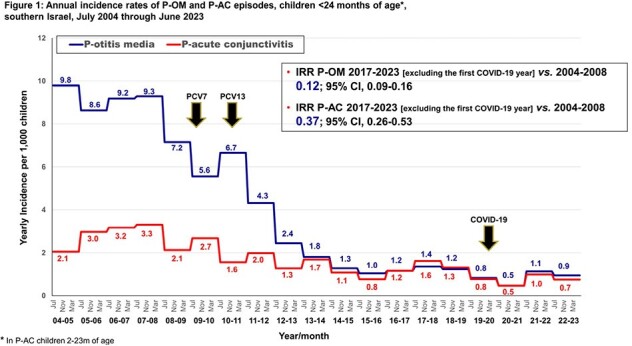

Annual incidence rates of P-OM and P-AC episodes, children <24 months of age*,

southern Israel, July 2004 through June 2023

**Methods:**

A retrospective analysis of two prospective, population-based surveillance projects on OM (Ben-Shimol CID 63:611, 2016) and AC (Dagan CID 72:1200, 2021) in southern Israel. All episodes in children < 24m with OM and/or AC with cultures in southern Israel have been included. All middle-ear fluid (MEF) and conjunctival cultures in the region are processed in a single laboratory of the hospital where ∼95% of all children are born and treated, permitting incidence calculations. The current analysis covers all pneumococcal OM and AC (P-OM, P-AC) from July 2004 through June 2023.

**Figure:**
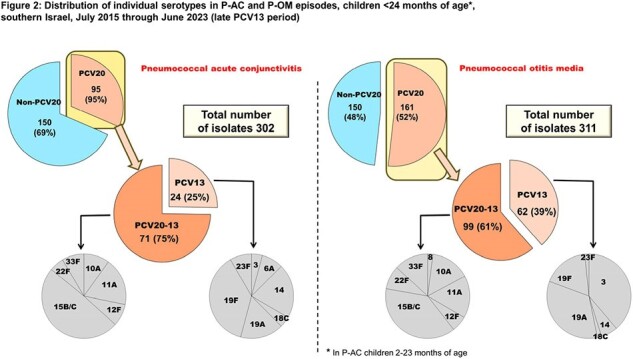

Distribution of individual serotypes in P-AC and P-OM episodes, children <24 months of age*,

southern Israel, July 2015 through June 2023 (late PCV13 period)

**Results:**

A total of 2,312 and 1,039 episodes of P-OM and P-AC, respectively, were observed. The P-OM and P-AC annual rates are presented in **Figure 1**. The respective incidence rate ratios (95% CI) for 2017-2023 (excluding Corona year 2020-2021) *vs*. 2004-2008 were 0.12 (0.09-0.16) and 0.37 (0.26-0.53). During the stable PCV13 period (2015-2023), 32% and 24% of all P-OM and P-AC, respectively, were PCV20 serotypes **(Figure 2)**. In both diseases, the PCV13 serotypes 19A, 19F, 3, and 14, and the PCV20-non-PCV13 serotypes 10A, 11A, 33F, and 15 B/C, predominated. PCV13 serotypes 1, 4, 5, 6A, 6B, 7F, 9V, 18C, 23F (grouped) constituted only 0.6% and 1.7% of all P-OM and P-AC, respectively.

**Conclusion:**

1) PCV13 implementation resulted in a rapid and steep decline in both P-OM and P-AC rates, stabilizing within 3-4 years; 2) during the late PCV13 period, only 4 PCV13 serotypes persisted: PCV7 serotypes 19F and 14, and PCV13 non-PCV7 serotypes 3 and 19A; and 3) the non-PCV13 PCV20 serotypes constitute approximately one quarter and one third of P-AC and P-OM, respectively, in children < 24m in southern Israel. Implementation of PCV20 has the potential to add benefit against pneumococcal mucosal disease.

**Disclosures:**

**Ron Dagan, Professor MD**, GSK: Advisor/Consultant|GSK: Honoraria|MedIMmune/AstraZeneca: Grant/Research Support|MedIMmune/AstraZeneca: Honoraria|MSD: Advisor/Consultant|MSD: Grant/Research Support|MSD: Honoraria|Pfizer: Advisor/Consultant|Pfizer: Grant/Research Support|Pfizer: Honoraria|Sanofi Pasteur: Honoraria

